# Penile Ulcer Atra Related in Patient with Acute Promyelocytic Leukemia

**DOI:** 10.4084/MJHID.2012.054

**Published:** 2012-08-09

**Authors:** Irfan Yavasoglu, Mustafa Unubol, Gokhan Sargin, Gurhan Kadikoylu, Zahit Bolaman

**Affiliations:** 1 Associate Professor, Internist, Hematologist, Adnan Menderes University Medical Faculty, Division of Hematology, Aydin, Turkey; 2 Fellow in Endocrinology, Internist, Adnan Menderes University Medical Faculty, Division of Diabetes, Metabolism and Endocrinology, Aydin, Turkey

**Dear editor,**

Addition of all-transretinoic acid (ATRA) to antracyclines has been a turning point in the treatment of acute promyelocytic leukemia raising the worldwide complete remission rate to more than 80%. However, a few new side effects have emerged.[1] Among side effects, differentiation syndrome (DS), formerly known as retinoic acid syndrome, is the main life-threatening complication of therapy with differentiating agents [all-trans retinoic acid (ATRA) or arsenic trioxide (ATO)].[1] Mild skin side effects including dry skin, xerostomia, cheilitis are also frequent but are not of clinical importance; however, skin ulcerations, more frequently described at the scrotum, [1–4] may rarely occur.

Our patient, a 29-year-old man, presented with gingival bleeding and was diagnosed with acute promyelocytic leukemia through bone marrow and genetic assessment. He was started on cytosine arabinoside, idarubicin and 45 mg/m^2^ ATRA combination chemotherapy. On treatment day 14, a painless ulcer with a diameter of approximately 1 cm, with an erythematous base emerged on the patient penis (Figure 1). His history included oral aphthae and acne on his back. The patient’s anti-HIV and VDRL tests were negative and eye examination was normal. The Pathergy test performed for Behcet’s disease was negative. Culture for haemophilus ducreyi was negative, as well ASCA Test for Saccharomyces Cervisiae Antibody. Since the ulcer occurred during ATRA therapy, without evidence for other possible diagnoses such as bacterial or viral infection, the genital ulcer was considered ATRA-related and ATRA was discontinued. On day 20, the penile ulcer healed with a scar. ATRA was then restarted without further complications.

While a limited number of scrotal ulcer related to ATRA use was reported in the literature [1–4], to our knowledge, penile ulcers have not been reported so far. Thus, penile ulcer may develop during ATRA therapy and can heal after stopping ATRA.

## Figures and Tables

**Figure 1:**
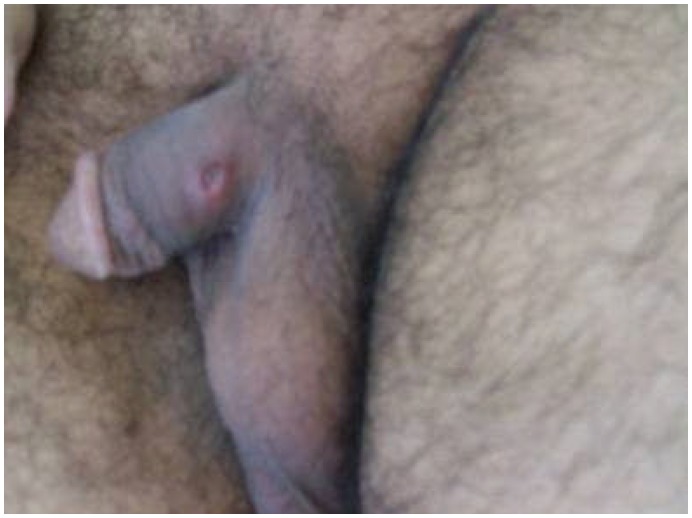
Painless penile ulcer of 1 cm diameter, on an erythematous base.

## References

[1] Jeddi R, Ghédira H, Ben Amor R, Ben Abdennebi Y, Karima K, Mohamed Z, Ben Neji H, Aissaoui L, Ben Lakhal R, Ben Salah N, Menif S, Belhadjali Z, Ben Abid H, Gouider E, Hafsia R, Saad A, Fenaux P, Meddeb B (2011). Treatment of Acute Promyelocytic Leukemia with AIDA Based Regimen. Update of a Tunisian Single Center Study. Mediterr J Hematol Infect Dis.

[2] Esser AC, Nossa R (2000). All-trans-retinoic acid-induced scrotal ulcerations in a patient with acute promyelocytic leukaemia. J Am Acad Dermatol.

[3] Charles KS, Kanaa M, Winfield DA, Reilly JT (2000). Scrotal ulcerations during all-trans retinoic acid (ATRA) therapy foracute promyelocytic leukaemia. Clin Lab Haematol.

[4] Ramzi J, Hend BN, Lamia A, Raihane BL, Hela BA, Zaher BA, Balkis M (2007). Scrotal ulcerations during all-transretinoic acid therapy for acute promyelocytic leukemia. Ann Hematol.

